# Psychometric Validation of the Caregiver Preparedness Scale in a Population-Based Sample

**DOI:** 10.3390/nursrep16040115

**Published:** 2026-03-31

**Authors:** Jiri Remr

**Affiliations:** INESAN (Institute for Evaluations and Social Analyses), Sokolovská 351/25, 18600 Prague, Czech Republic; jiri.remr@inesan.eu

**Keywords:** caregiver preparedness, Caregiver Preparedness Scale, psychometric validation, informal caregiver, population-based survey, known-groups validity

## Abstract

**Background/Objectives**: In the context of nursing research and interventions, caregiver preparedness emerges as a pivotal concept. Informal caregivers play a central role in providing older adults with the vital nursing and social support they require. The present study evaluated the psychometric performance of the Caregiver Preparedness Scale (CPS) and tested the hypothesis that CPS scores differentiate between theoretically relevant known groups, including caregiving exposure and relationship-based indicators. **Methods**: A cross-sectional, face-to-face survey was conducted in June 2025 among the general population of Czechia. A total of 1024 interviews were included in the analysis. The sample was randomly split for exploratory factor analysis (EFA) and confirmatory factor analysis (CFA). The internal consistency of the scale was assessed using Cronbach’s α and McDonald’s ω, while inter-item associations were evaluated with Kendall’s tau-b. The known-groups validity was assessed through nonparametric group comparisons across caregiving exposure, relationship indicators within the caregiver–senior dyad, caregivers’ self-rated health, and their life satisfaction. **Results**: The CPS demonstrated high internal consistency (Cronbach’s α = 0.944; McDonald’s ω = 0.944), robust item–total correlations (0.730–0.863), and acceptable floor and ceiling effects. The EFA supported a dominant one-factor solution (eigenvalue = 5.749), which explained 71.9% of the variance and had strong loadings (0.750–0.894). The CFA demonstrated a good fit (RMSEA = 0.069, SRMR = 0.0155, CFI = 0.990, and TLI = 0.980) after allowing for a limited number of conceptually justified residual covariances. Known-groups analysis supported the sensitivity of the scale when the CPS scores were higher among primary (M = 25.30) and secondary (M = 22.73) caregivers in comparison to non-caregivers (M = 18.38). Moreover, statistically significant differences were observed among those who provided care during the past five years (M = 24.30) compared to those without such experience (M = 18.12). CPS scores also exhibited variation in relationship-focused indicators in the anticipated directions, and were lower among respondents reporting poorer health and lower life satisfaction. **Conclusions**: The study provided consistent evidence that CPS is a reliable, unidimensional measure with robust known-groups validity. The CPS can be regarded as a suitable research instrument for nursing research and for evaluating interventions aimed at supporting informal caregivers.

## 1. Introduction

In aging societies, the need for consistent and long-term support in the home environment is increasing due to chronic diseases, multimorbidity, and reduced independence and self-sufficiency among seniors [1,2]. In this context, informal caregivers from among family members are indispensable partners who bear a significant share of the responsibility for the care provided [3,4,5]. The scope of their responsibilities encompasses a wide range of tasks, including assistance with daily activities, monitoring health status, and coordinating health care and social services. Recent research indicates that individuals who are better prepared to provide such intensive care tend to achieve more favorable outcomes in the care [6,7,8]. Additionally, these individuals demonstrate enhanced ability to cope with the demands of caregiving [9,10,11], exhibit reduced levels of depressive symptoms related to the care [12,13], and generally attain a higher quality of life [14]. Therefore, it is desirable to enhance and cultivate the preparedness of caregivers through targeted interventions, such as education and counseling. The initial step in this process is to ascertain the level of caregiver preparedness.

In this context, caregiver preparedness emerges as a pivotal construct that typically refers to an individual’s subjective perception of their ability to cope with the practical, emotional, and organizational demands associated with caregiving [15,16,17,18]. Preparedness is conceptualized with respect to reciprocity and other factors contributing to caregivers’ adaptation to their caregiving role [4,19,20]. Research has demonstrated that care outcomes are contingent not solely on objective circumstances, but also on the caregivers’ perceived ability to cope with the expectations and burden imposed upon them. Empirical evidence has demonstrated a relationship between preparedness and caregiver burden [10,11,14]. Consequently, caregivers who feel better prepared tend to cope more effectively with challenging situations [21]. Current knowledge suggests that psychosocial education, supportive counseling, and self-care programs can improve caregiver preparedness [22,23,24]. In addition, many studies have also linked higher caregiver preparedness to more favorable caregiver psychological functioning, including higher resilience and lower distress/burden [15,25,26]. These results position preparedness as a suitable goal of caregiver support interventions.

Caregiver preparedness differs from other constructs in this field. A plethora of research instruments have been developed to evaluate the well-being of caregivers and their capacity to provide care, to measure the caregiver burden, or to quantify caregiver stress, e.g., [7,10,12,27]. However, these instruments do not directly evaluate the perceived preparedness to meet care demands. Another related construct is caregiver self-efficacy, which is typically measured using domain-specific self-efficacy scales such as the Revised Scale for Caregiving Self-efficacy [28]. Such measures identify confidence in specific areas rather than providing a general assessment of an individual’s preparedness for the caregiving role.

To assess the caregiver preparedness, the Caregiver Preparedness Scale (CPS) was developed as a research tool to measure caregivers’ perceived preparedness in key domains of care [15]. These domains cover preparedness for physical and emotional care, service coordination, stress management, response to emergencies, orientation in the healthcare system, and overall preparedness [15]. Psychometric studies conducted among caregivers have confirmed its unidimensional structure and adequate reliability [29,30,31,32,33]. The CPS has been utilized in various care contexts in the past, which has enabled multiple comparisons. For instance, studies in oncology have examined care models in which preparedness predicted different outcomes for caregivers during the treatment [31,32,34,35]. These studies recommended that assessments consider not only the demands of care but also the quality of the relationship between caregiver and care recipient. In the domain of palliative care, numerous studies, e.g., [9,11,36,37,38], have examined the significance of preparedness, frequently in conjunction with other constructs such as competence or rewards. The CPS has been used in research engaging caregivers of stroke survivors [39,40], hemodialysis patients [41], and individuals with chronic diseases [30]. These results supported the hypothesis that the CPS can be used in contexts outside the original geriatric setting.

In addition to these studies, other research has expanded the scale’s use among individuals providing care in various settings (e.g., [31,41,42,43,44]). However, research regarding the scale’s performance among individuals who may soon assume a caregiving role is scarce. Furthermore, to the best of our knowledge, the CPS has not been validated in Czech society. Therefore, the objective of this study was to validate the Czech version of the CPS on a sample of the general population and to evaluate its psychometric properties. In addition to examining reliability and factor validity, we employed known-groups validity based on theoretically pertinent indicators that reflected the participation in care and characteristics of the relationship between the caregiver and care recipient. The scale’s functionality encompasses the theoretical prediction of disparities among designated groups, contingent on their engagement in caregiving activities and the nature of their relationships with the care recipient.

The rationale for the present study extends beyond the linguistic adaptation of an existing instrument. Family caregiving is often assumed in unexpected and uncertain circumstances, when relatives must rapidly manage a range of practical tasks, emotional support, and navigation of health and social services [4,16,21]. In this regard, preparedness is not only relevant among individuals already engaged in caregiving but also among those who may become caregivers in the near future. A population-based validation thus facilitates the examination of preparedness as anticipated or experienced caregiving, which is of particular importance for gerontological nursing, discharge planning, and caregiver support. The present study aims to contribute to psychometric evidence and substantive understanding of which conditions are associated with preparedness for informal family care. To that end, the study tests the CPS in a general-population sample and links it to caregiving exposure, relationship characteristics, self-rated health, and life satisfaction. The following hypotheses were formulated:

**H1.**  *The CPS demonstrates a unidimensional structure*.

**H2.**  *The CPS demonstrates adequate internal consistency, as substantiated by both Cronbach’s α and McDonald’s ω values*.

**H3.**  *The psychometric properties of the CPS demonstrate a good fit of the unidimensional model to the empirical data*.

## 2. Materials and Methods

### 2.1. Study Design

The study was performed as a quantitative, cross-sectional survey based on face-to-face interviewing among the general population of Czechia. An address-based sampling was employed to identify the dwellings within primary territorial units based on census data, i.e., the most accurate and comprehensive sampling frame available. Identified dwellings were then visited by the trained interviewers who identified the appropriate respondents within each household with the use of Kish tables [45].

### 2.2. Participants and Procedures

The research protocol was conducted in accordance with the ethical principles outlined in the Declaration of Helsinki [46], and approval was obtained from the INESAN Research Ethics Board (IREBA/2025/506; 6 May 2025). The data collection process involved the administration of face-to-face interviews, when 1937 individuals from the general population were invited to participate in June 2025. From this sample, 1071 interviews were completed, yielding a response rate of 55.3%, which is defined in accordance with the AAPOR-5 criteria. Prior to each interview, the interviewers provided the respondents with the following information: interviewing routines and procedures, approximate duration of the interview, confidentiality of responses, voluntary participation, beneficence, purpose of the study, and other details of the fieldwork ethics. Subsequently, the dataset was subjected to a quality check, resulting in the elimination of 47 cases due to data incompleteness. The final dataset used for psychometric analyses then contained 1024 cases.

The total sample, comprising 1024 respondents, was split into two subsamples: the first subsample was allocated for EFA, while the second subsample was designated for CFA. The resulting subsamples exhibited notable similarities in terms of gender, age distribution, and size of settlement, as evidenced in Table 1.

Table 1 illustrates that the sample demonstrated a high degree of similarity to the population with respect to gender, age, and settlement size, with deviations generally within 1–2 percentage points. Accordingly, while the threat of non-response bias persists, the attained sample can be regarded as adequately representative of the fundamental characteristics utilized in the sampling design.

### 2.3. Translation and Adaptation of the Scale

For the present study, the wording of individual items was adapted to refer to informal family care provided to an older adult family member. This minor adaptation enabled an examination of preparedness across a sample with varying caregiving experiences, encompassing those currently providing care and even those anticipating a caregiving role. The decision to focus on older adult family members was a deliberate one. The original conceptualization of preparedness emerged in the context of family care and gerontological nursing [15], and the present study was designed to preserve that substantive anchor. Secondly, the demands of caregiving vary across different care-recipient groups. Focusing the analysis exclusively on older adults serves to enhance conceptual homogeneity. Thirdly, the emphasis on older family members aligns with the predominant context in which preparedness is evaluated in nursing research, thereby enhancing the interpretability of known-groups comparisons in relation to caregiving exposure, relationship quality, and personal resources [16,18].

To ensure linguistic accuracy and conceptual equivalence, the translation and adaptation of the scale followed a multi-step procedure proposed for this purpose [47,48]. In this regard, two independent translations from English into Czech were conducted. Then, a panel of experts compared and reconciled the two versions, focusing on semantic, idiomatic, and conceptual equivalence. Later, a translator who was not involved in the forward translation completed a back-translation from Czech into English. Finally, the back-translated version was compared with the original instrument, and all discrepancies were resolved to ensure the appropriate meaning of each item and to avoid unintended bias [49,50]. The reconciliation of the two forward translations was undertaken by an expert panel consisting of three members with expertise in gerontological research, informal caregiving, and survey methodology. The selection of panel members was conducted purposively, with consideration given to their expertise in caregiving, proficiency in adapting questionnaires, and fluency in both Czech and English. In addition to semantic, idiomatic, and conceptual equivalence, the panel explicitly considered the relevance of each item, paying particular attention to the comprehensibility of caregiving terminology in Czech, the appropriateness of item wording for informal family care, and the applicability of the items to both current and prospective caregivers.

The pre-final version of the instrument was pilot-tested using one-to-one cognitive interviews grounded in verbal probing techniques [51,52]. A purposive sample of 11 participants was selected to ensure variation in gender, age, highest achieved education, and caregiving experience. Following the completion of the questionnaire, participants were asked to paraphrase individual items, explain how they interpreted the wording, describe how they arrived at their answers, and comment on the suitability of the response categories. A detailed examination was conducted, focusing on all four stages of the response process: item comprehension, information retrieval, the judgment underlying the selected response, and response formatting, including the risk of socially desirable responding. The interview notes were subjected to a thorough review, with only minor revisions introduced in four items where recurrent comprehension and response format issues were identified. The conceptual structure of the instrument was maintained in its entirety, with no items being removed.

### 2.4. Measures

#### 2.4.1. CPS

The CPS included eight items, each rated on a 5-point Likert-type scale, reflecting the extent to which respondents feel prepared to provide care. In accordance with previous applications, the CPS evaluated perceived preparedness to address the fundamental demands of caregiving, encompassing practical, emotional, and organizational dimensions [15]. The response options ranged from 0 (not at all prepared) to 4 (very well prepared). The item scores were aggregated into a total CPS score, with higher values denoting a greater perceived preparedness. The theoretical range of this summated score was 0–32.

#### 2.4.2. Auxiliary Indicators for Known-Groups Validity

The known-groups validity tests whether the instrument discriminates between groups expected to differ on theoretical or empirical grounds [53,54]. In this study, it was assessed by comparing CPS scores across groups defined by (a) an exposure to caregiving, (b) quality of relationship between caregiver and care recipient, and (c) physical and mental capacities to perform the caregiving tasks.

(a) Exposure to caregiving

For the purpose of this study, the term “primary caregiver” was defined as a respondent who identified themselves as the main person currently responsible for providing regular informal assistance to an older family member. A “secondary caregiver” was defined as a respondent who provided supportive care but did not bear the main responsibility for care. Respondents who reported no current informal caregiving role were classified as non-caregivers. The indicator of prior caregiving experience referred to having provided informal family care to an older adult at any time during the previous five years, irrespective of caregiving status. Caregiver preparedness is largely a matter of personal experience. Its development is a process shaped by repeated engagement in caregiving tasks and interactions with the healthcare system and social services. Caregiver preparedness plausibly increases through experiential learning when caregivers accumulate care-related knowledge and skills over time [55]. Caregivers actually providing care have had opportunities to acquire skills, calibrate expectations, and refine coping routines [55]. Conversely, individuals without caregiving experience typically do not possess specialized knowledge and have had no opportunities to gain feedback and learn. Therefore, individuals who are providing care are expected to demonstrate higher levels of preparedness compared to those who are not caregivers. Building on these assumptions, we incorporated the current caregiving role (primary, secondary, or none) as an indicator of exposure to caregiving.

Moreover, caregiver preparedness is not solely influenced by the current caregiving practices. The long-term accumulation of knowledge, familiarity with care coordination, and confidence in managing practical and emotional demands (which can be described as “preparedness capital”) represent a resource for future care roles [16]. Therefore, an indicator of long-term caregiving experience was included as well to reflect the differences in preparedness capital. The assumption was that individuals with prior experience with caregiving perceive their preparedness to be higher compared to individuals without caregiving history and without their own experience in this regard.

(b) Relationship-focused indicators

The relational context of caregiving constitutes an integral component of the theoretical framework of caregiver preparedness. As is delineated in the literature, caregiver preparedness is associated with the quality of the relationship with the care recipient, which is understood as mutuality, reciprocity, emotional closeness, or relational tension [56]. Because informal caregiving is embedded in ongoing interpersonal relationships, the quality of this relationship is a key factor for caregiving appraisals and outcomes. Other studies show that higher relationship quality is associated with lower perceived burden and better psychological well-being, whereas conflictual or ambivalent relationships are associated with greater strain [56,57]. Reciprocity and perceived balance have also been linked to caregiver perceived burden and well-being, consistently with equity and social exchange perspectives [19,57]. While the relationship quality is multifaceted and a single indicator cannot adequately capture its merit, the present study incorporated a range of four indicators to assess relationship quality. These indicators are (a) overall sympathy, (b) the extent to which the relationship with the care recipient meets expectations. These indicators capture the emotional and cognitive background of caregiver–care recipient bonds. Two other indicators reported (c) intended avoidance of contact, which has been linked with significant tension, emotional distance, and conflict, and (d) estimated amounts of problems and flaws in the relationship with the care recipient. It was assumed that caregivers in high-quality relationships with care recipients demonstrate higher preparedness than individuals with problematic relationships. For the relationship-focused indicators, the category “neither, nor” was interpreted as a neutral response. These indicators were retained separately because they were intended to assess different facets of relationship quality, including positive appraisal (liking), congruence with expectations, relational strain, and desire for distance. Although related, these dimensions are not interchangeable, and together they provide a more comprehensive perspective on the relational context within which preparedness is formed and appraised [19,56,57].

(c) Self-assessment of health and life satisfaction

Caregiver preparedness also encompasses the perceived capacity to care. In this respect, self-rated health is a useful indicator of functional capacity and perceived vitality that are important predispositions to caregiving [58]. Poorer health may reduce the ability to perform instrumental caregiving demands and may limit coping resources for care-related stress [26]. Moreover, life satisfaction as a cognitive evaluation of one’s life circumstances is commonly used in population research [59]. In prior studies, higher life satisfaction has been associated with greater optimism, better perceived control, and predicted more adaptive coping, whereas exposure to major stressors and resource constraints tends to reduce life satisfaction over time [60]. Therefore, it was assumed that individuals with better self-rated health demonstrate higher preparedness, and similarly, those who have higher life satisfaction perceive their preparedness to be higher.

### 2.5. Data Analysis

In order to support robust scale validation, as emphasized by numerous studies, e.g., [61], the sample was randomly divided into two subsamples for sequential analyses: one for exploratory factor analysis (EFA; n = 512) and the other for confirmatory factor analysis (CFA; n = 512). Given the number of CPS items (8), the subsample sizes provided adequate subject-to-item ratios for the intended analyses [62,63]. The characteristics of the sample and both subsamples (i.e., gender, age, and size of settlement) are reported in Table 1, alongside the Czech population data from the last census to contextualize the representativeness.

The data underwent rigorous analyses to assess item performance, scale reliability, factorial validity, and known-groups validity. Initially, item-level descriptive statistics were computed for each of the CPS items and for the total CPS score, including means, standard deviations, and distributional diagnostics [64,65]. Floor and ceiling effects were evaluated as the proportion of respondents scoring the minimum/maximum possible total score; these effects were considered negligible if less than 15% of respondents achieved the lowest or highest score [66]. With regard to the ordinal origin of the items, the observed distributional departure from normality, and the unequal group sizes across some comparisons, all inferential analyses of between-group differences were conducted using nonparametric procedures. Consequently, the median was regarded as a reliable complementary indicator of central tendency. Given the nature of CPS items, the inter-item associations were summarized using Kendall’s tau-b (τ_b), which is effective when associations are monotonic under ordinal measurement because it accounts for tied ranks [67].

The internal consistency of the scale was evaluated using Cronbach’s α and McDonald’s ω [68,69] to provide complementary indices of reliability under potentially non-tau-equivalent item structures. The examination of item discrimination utilized corrected item–total correlations and the alpha if item deleted to identify possible items that contributed marginally to the overall scale. Furthermore, composite reliability (CR) and average variance extracted (AVE) were employed to assess the measurement quality at the latent level.

The dimensionality and factorial validity of the scale were evaluated using a split-sample approach. The study employed EFA to examine the CPS’s underlying structure. Prior to factor extraction driven by principal axis factoring (PAF), sampling adequacy and factorability were assessed using the Kaiser–Meyer–Olkin (KMO) measure and Bartlett’s test of sphericity [70]. The factor retention process was guided by the eigenvalues, and its efficacy was verified through the examination of the scree plot [71,72]. In light of the prevailing single-factor solution, the implementation of rotation was deemed unsuitable.

Subsequently, the CFA was executed in the second subsample to evaluate the model proposed by the EFA. In this regard, the estimation of CFA models used maximum likelihood estimation in AMOS [73,74]. Examination of univariate item distributions (skewness and kurtosis) and multivariate normality diagnostics was conducted. Given the ordinal nature of items, bootstrap standard errors and bias-corrected confidence intervals were employed, with 2000 replications. The evaluation of model fit was conducted by employing the absolute and incremental indices, encompassing measures such as Bollen–Stine bootstrap test, root mean square error of approximation (RMSEA), standardized root mean squared residual (SRMR), goodness of fit index (GFI), comparative fit index (CFI), Tucker–Lewis index (TLI), and normed fit index (NFI) [75]. In accordance with the established guidelines [76], the CFI/TLI values ranging from 0.90 to 0.95 and the RMSEA/SRMR values less than 0.08 were interpreted as suggesting an acceptable to good fit. In instances where theoretical justifications consistent with prior CPS validations, e.g., [31,32,41], were met, limited residual covariances between conceptually similar items were considered as a means to enhance model fit [77].

The known-groups validity was assessed by testing the hypothesis that CPS scores would differ across the aforementioned auxiliary indicators, i.e., caregiving exposure, relationship-related indicators, self-rated health of caregivers, and caregivers’ life satisfaction. Given that the outcome is predicated on ordinal item responses and the presence of multiple grouping variables engenders disparate group sizes, between-groups comparisons were undertaken by means of nonparametric tests. For multi-category indicators, the Kruskal–Wallis H test was employed, while the Mann–Whitney U test was utilized for binary indicators. Effect sizes are reported as epsilon-squared (ε^2^) for Kruskal–Wallis tests and Cliff’s delta (δ) for Mann–Whitney tests. Statistical significance was evaluated at conventional levels (*p* < 0.05 and *p* < 0.01). All analyses were conducted using IBM SPSS Statistics version 26 (IBM Corp., Armonk, NY, USA) and AMOS version 24.

## 3. Results

### 3.1. Item Analysis and Reliability

An examination of item-level descriptive statistics and reliability indicators was conducted in the EFA subsample (n = 512). As illustrated in Table 2, the item means ranged from 2.11 to 2.73, indicating mid-level perceived preparedness across domains. The highest mean was observed for preparedness to find out and set up services (item C: M = 2.73, SD = 1.21) and preparedness to address emotional needs (item B: M = 2.67, SD = 1.11). Lower mean values were identified for preparedness to handle emergencies (item F: M = 2.11, SD = 1.21), coping with caregiving-related stress (item D: M = 2.18, SD = 1.25), and for overall preparedness to care (item H: M = 2.15, SD = 1.25).

The results indicated that the total CPS score ranged from 0 to 32, and the mean score was found to be 19.06, with a standard deviation of 8.203 and a median of 19. The CPS score distribution exhibited deviations from normality, as indicated by negative skewness (−0.431) and kurtosis (−0.291). Despite the deviation of the CPS total score from strict normality, the mean and standard deviation were maintained for descriptive purposes, as the total score constitutes a summated index with a readily interpretable metric. Moreover, these statistics enable comparison with previous CPS studies. The floor and ceiling effects were low, with the floor effect being 4.2% and the ceiling effect 7.7%. These results suggest that the scale demonstrates adequate variance, sensitivity across the entire range of values, and a low accumulation of data around the extreme values [66]. The internal consistency of the scale was high when Cronbach’s alpha and McDonald’s omega both achieved 0.944. As indicated by alpha if item deleted, removing any item would not improve the overall reliability of the scale [78] because in all such occasions, the alpha would be lower than for the full 8-item scale (0.932 to 0.941). Additionally, corrected item–total correlations were uniformly high (0.730–0.863), indicating strong item discrimination.

The associations between items, measured by Kendall’s τ_b, were consistently positive and statistically significant (all *p* < 0.01); values ranged from 0.476 to 0.735 (see Table 3 for details), supporting coherence without suggesting redundancy.

### 3.2. Factor Structure

EFA was conducted on the first subsample. The sampling adequacy was high (Kaiser–Meyer–Olkin = 0.927), and Bartlett’s test of sphericity was significant (χ^2^ = 3477.509, df = 28, *p* < 0.001), indicating that the inter-item correlation matrix was factorable. The EFA yielded a clear unidimensional structure, with all eight items loading strongly onto a single common factor. As demonstrated in Table 4, a one-factor solution was extracted with a predominant one factor (eigenvalue = 5.749), which accounted for 71.9% of the variance. The analysis revealed that the standardized loadings (F1) ranged from 0.750 to 0.894, and the communalities (h^2^) varied from 0.563 to 0.800. These results suggest that a significant portion of the observed variability among the items can be attributed to a general construct of preparedness. Convergent validity was examined using composite reliability (CR) and average variance extracted (AVE) following standard recommendations [79]. In this respect, model-based convergent indicators demonstrated robust performance, exhibiting high composite reliability (CR = 0.94) and average variance extracted (AVE = 0.68), thereby further substantiating a coherent latent preparedness factor.

### 3.3. Model Confirmation

CFA was conducted on the second subsample. Initially, a one-factor model with uncorrelated residuals was tested. The model fit was considered suboptimal, as indicated by incremental indices (CFI = 0.936, TLI = 0.910, and NFI = 0.931), while the SRMR was found to be low (0.0403). Furthermore, the absolute fit indices indicated a considerable misfit (RMSEA = 0.146), and the GFI was marginally below the conventional cutoff (GFI = 0.899). Despite the imperfect fit, the standardized factor loadings were strong (ranged from 0.76 to 0.87), and the latent factor explained a significant amount of the variance (R^2^ = 0.58–0.76), supporting the hypothesis of a single preparedness factor.

**Figure 1:**
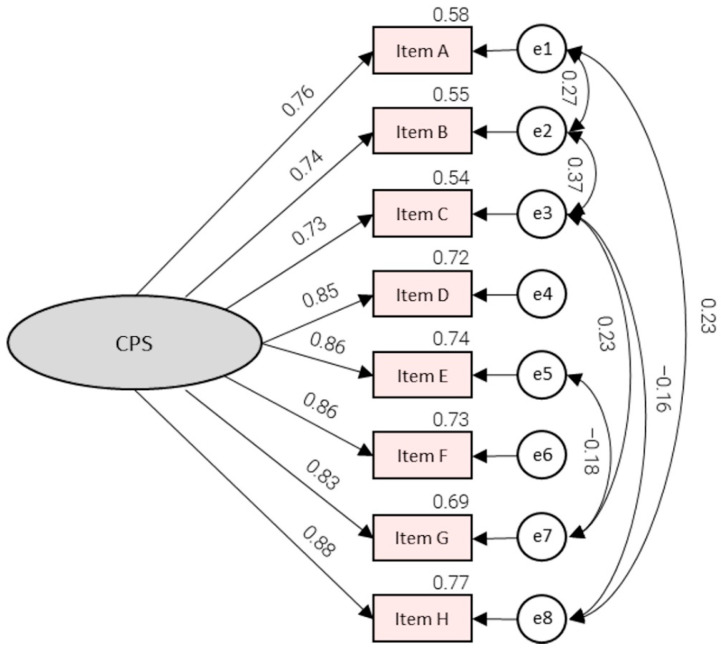
CPS confirmatory factor analysis (improved model).

Figure 1 presents an improved one-factor model that accommodates residual covariances among conceptually close items. The absolute and incremental indices presented in Table 5 met or even exceeded the usual criteria in the improved model (RMSEA = 0.069, SRMR = 0.0155, GFI = 0.978, CFI = 0.990, TLI = 0.980, and NFI = 0.986). The standardized loadings exhibited a consistent pattern, maintaining a range of 0.73 to 0.88, while the item R^2^ values demonstrated a similar trend, ranging from 0.54 to 0.77. These results suggest that the enhanced compatibility was attained without compromising the one-factor structure. Residual covariances were added only when modification indices were substantial, and the paired items shared specific content beyond the overall preparedness factor, thereby reducing the risk of overfitting. The residual covariances encompassed links among proximate preparedness domains (see A–B, B–C, E–G, C–G, C–H, and A–H), which aligned with an idea of minor local dependencies rather than multidimensionality.

### 3.4. Known-Groups Validity

The known-groups validity assessment corroborated the CPS’s capacity to differentiate preparedness based on caregiving exposure and other theoretically relevant circumstances. Table 6 shows significant variation of CPS scores according to the caregiver role (Kruskal–Wallis H = 30.880, *p* < 0.001) when primary caregivers demonstrated the highest CPS score (M = 25.30, SD = 5.502), followed by secondary caregivers (M = 22.73, SD = 5.637). Conversely, respondents who were not providing informal family care exhibited significantly lower preparedness (M = 18.38, SD = 8.217). A similar pattern emerged for caregiving experience when statistically significant discrepancy was observed in the preparedness reported by individuals who had previously provided informal care in comparison to those lacking such experience (Mann–Whitney U = 9207.5, *p* < 0.001). Individuals with prior caregiving experience exhibited higher preparedness compared to those without such experience (M = 24.30, SD = 5.905 vs. M = 18.12, SD = 8.208).

Relationship-focused indicators showed substantial disparities in the anticipated direction. The caregivers’ perceived preparedness exhibited a significant positive correlation with the extent to which their relationship with the care recipient met expectations (H = 88.852, *p* < 0.001; “yes” M = 23.19 vs. “neither, nor” M = 17.33 vs. “not” M = 11.58) and with the caregivers’ reported sympathy (H = 67.239, *p* < 0.001; “yes” M = 22.19 vs. “neither, nor” M = 16.18 vs. “not” M = 10.18). Conversely, preferred non-interaction with the senior was associated with reduced preparedness (H = 80.213, *p* < 0.001), and the indicator of relational strain significantly differentiated the preparedness among respondents reporting “many problems” from that among those who mentioned only a few flaws (H = 73.943, *p* < 0.001). In this respect, the CPS mean values were as follows: “many problems” (M = 12.02), “some problems” (M = 16.60), and “only a few problems” (M = 22.38).

On top of that, respondents who reported their poor health demonstrated lower preparedness compared to those who reported good health (Mann–Whitney U = 12,317.0, *p* = 0.001). The mean value was 16.17 in contrast to M = 19.81 in the good health category. A similar result was observed for life satisfaction (H = 12.200, *p* = 0.002), with lower preparedness among dissatisfied respondents (M = 15.00) compared with satisfied individuals (M = 19.80).

## 4. Discussion

The objective of the present study was to assess the validity of the CPS and to provide a comprehensive assessment of its psychometric characteristics on a nationwide sample of the general population. The results indicated that the CPS is a reliable instrument for assessing caregiver preparedness. Specifically, the scale demonstrated very good internal consistency and strong item discrimination. EFA identified a predominant one-dimensional structure. Further evidence demonstrated that the one-dimensional model can adequately reproduce the observed covariance structure when minor, theoretically justifiable local dependencies between several items were considered. Furthermore, the CPS demonstrated robust known-groups validity, effectively differentiating between individuals with varying degrees of engagement in caregiving and reflecting the quality of the relationship between caregivers and care recipients, and personal well-being. These results support the notion that the CPS can be utilized as a reliable measure within the domains of informal family care and gerontological nursing.

The CPS also demonstrated adequate performance at the item level. The internal consistency estimates were found to be excellent, and the correlations of items with the total score were also high. The results indicated that each item contributes to the overall score and is commensurate with the construct. The absence of evidence indicating that any item compromised the integrity of the scale supported the retention of the full eight-item version. The low floor and ceiling effects indicated that the CPS can differentiate respondents with different levels of perceived preparedness. This is of particular importance because the instrument can be employed in the screening process, which might include individuals with low and high levels of preparedness. It is also important for the use of the scale in program evaluations, where ceiling effects could mask improvements following caregiver support interventions.

A high degree of internal consistency may indicate the redundancy of items or the narrowed focus on a limited part of a whole continuum. However, in this case, several results mitigate this concern. Initially, the distribution of the scale exhibiting low floor and ceiling effects signifies adequate data dispersion. Secondly, the associations between items were consistently positive and moderate to strong, though not too strong to indicate interchangeability [80]. Thirdly, the mean scores indicated significant variations in perceived preparedness based on the caregiving context. The respondents indicated a lower level of preparedness for acute or high-risk situations (e.g., handling emergencies and coping with stress) in comparison to their preparedness for more routine or structured tasks (e.g., providing services or addressing emotional needs). This variation is not only plausible but also consistent with the caregiving experience, which is often characterized by uncertainty, crisis management, and emotional stress [16,37,81,82]. The results thus support the interpretation that the CPS captures an overall preparedness rather than the single skills.

In the initial specification of CFA, the standardized factor loadings were strong, and the latent factor explained a significant portion of the variance in the items. This result is consistent with a robust construct of overall preparedness; however, global fit indices indicated that the one-factor model with uncorrelated residuals did not fully reproduce the observed covariance structure. This is common in short scales, where items may exhibit shared variability due to their similarity in wording, the overlapping content, or factual interconnections [77,83]. The incorporation of a limited number of correlated residuals in the improved model led to a better overall fit of the model, while maintaining the single-factor structure and the robustness of factor loadings. The residual covariances can be interpreted as substantially meaningful and not merely as statistical artifacts. It has been demonstrated that items reflecting closely related competencies, e.g., meeting physical and emotional needs or organizing services, may activate similar coping strategies. Consequently, these items may produce shared variance beyond the general factor. Furthermore, the general preparedness item (item H) exhibited correlation with numerous items because respondents may have interpreted it as a summary judgment derived from these domains. Such interpretation aligns with the prevailing understanding of preparedness in real-world caregiving contexts, where preparedness is understood as a general sentiment associated with specific demands. On top of that, the presence of multiple residual correlations does not compromise the interpretability of the CPS as a single-factor instrument.

Known-groups analyses have demonstrated that the CPS performs as anticipated with respect to engagement in care. The highest level of preparedness was observed among primary caregivers, a medium level among other caregivers, and the lowest among those who do not provide care. Furthermore, recent experience with care was found to be associated with higher levels of preparedness. These gradients align with the theoretical justification of preparedness, which posits that direct engagement in caregiving, repeated contact with health care and social services, and the accumulation of experience lead to an increased perception of preparedness [4,84].

A notable contribution of this study is the analysis of the associations between caregiver preparedness and the quality of the relationship between caregiver and care recipient. The results indicated that respondents who characterized their relationship as positive demonstrated a significantly higher level of preparedness. In contrast, relationship tensions were associated with lower preparedness. These results align with the conclusions of other studies that underscore the pivotal role of the relationship in caregivers’ adaptation to their role and their perceived ability to cope with the demands of caregiving [56,57,85]. The results further underscore that preparedness is not only an individual attribute reflecting knowledge or skills, but rather, it is rooted in a relational context that can either support or limit caregiving. Tensions in the relationship with the care recipient have been demonstrated to exert a deleterious effect on caregivers’ self-efficacy and their perceived preparedness. The results indicated the usefulness of an integrated approach that combines the caregivers’ knowledge and skills with effective communication, expectation management, and relational support.

The study’s results also indicate a correlation between preparedness and self-rated health as well as life satisfaction. Respondents who assessed their health as poorer exhibited lower preparedness, thereby corroborating the hypothesis that physical or functional limitations may diminish confidence in coping with practical caregiving tasks and in the sustainability of the caregiving role. Lower life satisfaction was associated with lower preparedness, which may reflect psychosocial vulnerability [10,12,57] or cumulative stress [7,12]. In the context of nursing practice, these results underscore the necessity to customize interventions aimed at cultivating caregiver preparedness to encompass not only the care as such but also other skills such as self-care, planning, and gaining access to support resources and help. This is particularly important for caregivers with poorer health or lower psychosocial capacities.

Several limitations of the study should be acknowledged. Firstly, the cross-sectional design imposes limitations on the inferences that can be drawn regarding the potential predictive validity. Longitudinal research should be conducted to ascertain whether CPS scores can serve as a predictor of caregiver performance or capacity to cope with caregiver burden and stress. Secondly, while the scale has demonstrated its efficacy in this population sample, further research is necessary to assess measurement invariance across different population segments and to verify whether these groups actually differ in their level of preparedness. Thirdly, the CFA was based on a model that was improved by the inclusion of residual covariances. While this approach is theoretically substantiated and consistent with prior CPS validations [31,32,41,42,44], subsequent analyses could enhance the robustness of the conclusions by employing estimates that explicitly model ordinal indicators. Fourthly, it is appropriate to consider the possibility of social desirability bias. Because preparedness and relationship appraisals are socially valued characteristics and the survey was administered face-to-face, some respondents may have overstated their perceived preparedness or the quality of their relationship to conform to perceived norms. Although the CPS uses non-judgmental wording, future research could mitigate this risk by offering self-administered modes, emphasizing anonymity, and incorporating validation indicators such as behavioral proxies or informant reports. Fifthly, although the sample exhibited a high degree of correspondence with the distribution of the theoretical population with regard to key characteristics, minor deviations should be considered when interpreting the prevalence of preparedness. A final limitation concerns the heterogeneity of caregiving status in the sample. Because most respondents were not current caregivers, part of the variance captured by the CPS reflects anticipated rather than directly experienced preparedness. This is analytically valuable for understanding preparedness before caregiving begins, but it also limits direct comparability with studies based exclusively on active caregivers. Future research should therefore re-examine the factorial structure, measurement invariance, and predictive validity of the CPS separately in confirmed caregiver and non-caregiver samples, and preferably in longitudinal designs that follow transitions into caregiving.

The present study makes a unique contribution to the field by providing the first Czech-language validation of the CPS. The study’s comprehensive approach, encompassing both current caregivers and respondents who may assume caregiving roles in the future, refines the conceptualization of preparedness. It moves beyond a narrow definition of preparedness as a characteristic exclusive to those currently providing care, emphasizing an expanded capacity that encompasses future preparedness. The observed variations in caregivers’ preparedness, as determined by their current caregiving role and prior caregiving experience, lend support to the hypothesis that preparedness is shaped by exposure to care. The associations with relationship quality, health, and life satisfaction suggest that preparedness is embedded in broader relational and psychosocial contexts. In this sense, the study contributes substantive knowledge regarding the social and personal patterns of preparedness in a general population, extending beyond the scope of psychometric performance.

The present findings also have several implications for future research. The CPS has been demonstrated to be a suitable psychometric measure and an outcome indicator in studies evaluating caregiver support interventions. Future studies should test its longitudinal sensitivity to change, predictive validity in relation to caregiver burden, distress, and persistence in the caregiving role, and measurement invariance across subgroups defined by caregiving, age, education, and health status. The implementation of such studies would facilitate the elucidation of two primary research questions. Firstly, the study would ascertain whether the CPS performs consistently across various contexts. Secondly, the study would examine which factors contribute to or undermine the development of preparedness over time.

The findings are also pertinent to nursing education. The item-level pattern indicates that respondents perceived themselves to be less adequately prepared for emergencies and the stress associated with caregiving than for some more routine aspects of care. Consequently, educational support should not be confined to practical instructions but should also encompass stress management, communication with older relatives, service navigation, and realistic planning. In this context, the CPS can function as a brief screening and evaluation instrument, assisting educators in identifying specific domains of low preparedness and subsequently tailoring educational content accordingly.

The results of this study demonstrate the usefulness of systematic preparedness assessment in nursing administration, particularly in the domains of discharge planning, transitional care, community nursing, and caregiver support services. An association has been demonstrated between lower preparedness and the absence of caregiving experience, as well as poorer health, lower life satisfaction, and more strained relationships. Consequently, preparedness can be conceptualized not merely as a deficiency in skills but rather as an indicator of caregiving vulnerability. The utilization of the CPS by health and social policy administrators and policymakers has been demonstrated to facilitate the identification of groups exhibiting elevated risk. This identification process enables the prioritization of targeted psychoeducation and care coordination initiatives. Additionally, the evaluation of support services has been demonstrated to enhance the sustainability and quality of informal care for older adults.

## 5. Conclusions

The CPS was validated using a split-half sample with independently conducted exploratory and confirmatory factor analysis. The results indicated that the CPS offers a reliable evaluation of perceived preparedness to provide informal care. The results offered evidence that validates the CPS as a unidimensional construct.

The validity based on known-groups analysis led to enhanced interpretability of the scale. A significant differentiation in the CPS scores was observed according to engagement in caregiving, with current caregivers demonstrating higher levels of preparedness compared to individuals who were not caregivers. Furthermore, the CPS scores exhibited significant associations with relationship quality, which was consistent with established frameworks emphasizing relational resources and relational strain as pivotal determinants of caregiver adaptation.

The CPS was determined to be a concise, internally coherent, and psychometrically robust measure of perceived preparedness for informal family care. Beyond confirming the scale’s unidimensional structure and reliability, the present study demonstrated that preparedness varies systematically according to caregiving exposure, prior caregiving experience, relationship quality, self-rated health, and life satisfaction. These findings suggest that preparedness is not merely a technical competence, but rather a broader caregiving resource shaped by experiential, relational, and psychosocial conditions. Consequently, the CPS appears to be a suitable instrument for a variety of applications, including psychometric research, population surveys, needs assessments, discharge-planning routines, and evaluations of caregiver support interventions. Its implementation may assist nursing professionals and service providers in identifying individuals with inadequate preparedness at an earlier stage and in designing more targeted forms of educational, psychosocial, and organizational support.

## Figures and Tables

**Table 1:** Selected socio-demographic characteristics.

Variables		Total Population	Total Sample	EFA Sample	CFA Sample
Gender	Male	50.4%	50.0%	49.8%	51.0%
	Female	49.6%	50.0%	50.2%	49.0%
	Total	100.0%	100.0%	100.0%	100.0%
Age	15–29 years	19.4%	20.1%	20.6%	20.2%
	30–39 years	18.1%	17.8%	18.1%	18.1%
	40–49 years	21.0%	21.7%	20.3%	21.7%
	50–59 years	18.8%	17.6%	19.7%	18.9%	
	60–74 years	22.7%	22.9%	21.3%	21.1%
	Total	100.0%	100.0%	100.0%	100.0%
Size of settlement	Less than 1000 inhabitants	16.2%	16.9%	16.5%	16.8%
	1000 to 4999 inhabitants	22.4%	22.3%	22.1%	21.6%
	5000 to 19,999 inhabitants	19.5%	18.0%	18.7%	18.1%
	20,000 to 99,999 inhabitants	20.7%	20.1%	20.0%	20.4%
	100,000 inhabitants and more	22.3%	22.7%	22.5%	23.1%
	Total	100.0%	100.0%	100.0%	100.0%

**Table 2:** Descriptive statistics of the CPS and its items.

	N	Mean	SD	ITC	Alpha If Item Deleted
(a) How well prepared do you think you are to take care of your family member’s physical needs?	512	2.31	1.235	0.768	0.938
(b) How well prepared do you think you are to take care of his or her emotional needs?	512	2.67	1.105	0.730	0.941
(c) How well prepared do you think you are to find out about and set up services for him or her?	512	2.73	1.211	0.744	0.940
(d) How well prepared do you think you are for the stress of caregiving?	512	2.18	1.254	0.795	0.936
(e) How well prepared do you think you are to make caregiving activities pleasant for both you and your family member?	512	2.27	1.207	0.841	0.933
(f) How well prepared do you think you are to respond to and handle emergencies that involve him or her?	512	2.11	1.214	0.818	0.935
(g) How well prepared do you think you are to get the help and information you need from the health care system?	512	2.51	1.227	0.814	0.935
(h) Overall, how well prepared do you think you are to care for your family member?	512	2.15	1.254	0.863	0.932

**Table 3:** Correlation of CPS items.

	Item A	Item B	Item C	Item D	Item E	Item F	Item G	Item H
Item A	1.000							
Item B	0.544 **	1.000						
Item C	0.497 **	0.595 **	1.000					
Item D	0.594 **	0.506 **	0.515 **	1.000				
Item E	0.572 **	0.545 **	0.566 **	0.664 **	1.000			
Item F	0.609 **	0.479 **	0.476 **	0.655 **	0.694 **	1.000		
Item G	0.535 **	0.571 **	0.649 **	0.580 **	0.632 **	0.615 **	1.000	
Item H	0.683 **	0.567 **	0.523 **	0.694 **	0.672 **	0.735 **	0.690 **	1.000

Note: Kendall’s tau_b; ** correlation is significant at the 0.01 level.

**Table 4:** CPS exploratory factor analysis.

	N	F1	h^2^
(a) How well prepared do you think you are to take care of your family member’s physical needs?	512	0.793	0.629
(b) How well prepared do you think you are to take care of his or her emotional needs?	512	0.750	0.563
(c) How well prepared do you think you are to find out about and set up services for him or her?	512	0.766	0.587
(d) How well prepared do you think you are for the stress of caregiving?	512	0.822	0.676
(e) How well prepared do you think you are to make caregiving activities pleasant for both you and your family member?	512	0.869	0.756
(f) How well prepared do you think you are to respond to and handle emergencies that involve him or her?	512	0.847	0.717
(g) How well prepared do you think you are to get the help and information you need from the health care system?	512	0.841	0.708
(h) Overall, how well prepared do you think you are to care for your family member?	512	0.894	0.800

**Table 5:** CPS absolute and incremental indices.

	Bollen–Stine *p*-Value	RMSEA	SRMR	GFI	CFI	TLI	NFI
Original model	*p* < 0.001	0.146	0.0403	0.899	0.936	0.910	0.931
Improved model	*p* = 0.011	0.069	0.0155	0.978	0.990	0.980	0.986
Critical values		<0.08	<0.07	>0.95	>0.90	>0.95	>0.95

**Table 6:** Associations of the CPS with other indicators.

	%	Mean	SD	U/H	*p*-Value	*ε^2^/δ*
Respondent engagement in informal family care				30.880	<0.001 ^a^	0.06
Primary caregiver	7.9%	25.30	5.502			
Secondary caregiver	3.0%	22.73	5.637			
Not providing informal family care	89.1%	18.38	8.217			
Informal family care performed in the last 5 years				9207.500	<0.001 ^b^	–0.44
Yes	15.2%	24.30	5.905			
No	84.8%	18.12	8.208			
Does your relationship with senior meet your expectations?				88.852	<0.001 ^a^	0.24
Yes	60.4%	23.19	6.999			
Neither, nor	29.0%	17.33	6.310			
Not	10.6%	11.58	8.258			
Do you like the senior in your family?				67.239	<0.001 ^a^	0.18
Yes	74.1%	22.19	7.383			
Neither, nor	19.8%	16.18	5.922			
Not	6.1%	10.18	8.528			
How often do you wish you had no relationship with the senior?				80.213	<0.001 ^a^	0.22
Very often	17.5%	13.54	7.986			
Sometimes	13.4%	16.31	6.130			
Never	69.1%	22.74	6.968			
How many problems in the relationship with this senior do you have?				73.943	<0.001 ^a^	0.20
Many problems	11.4%	12.02	7.696			
Some problems	16.2%	16.60	6.366			
Only a few problems	72.4%	22.38	7.200			
Self-rated health of the respondent				12,317.000	0.001 ^b^	–0.24
Good	84.2%	19.81	7.925			
Bad	15.8%	16.17	8.510			
Overall life satisfaction of the respondent				12.200	0.002 ^a^	0.02
Satisfied	63.1%	19.80	8.217			
Neither, nor	31.0%	18.29	8.004			
Dissatisfied	6.0%	15.00	7.773			

^(a)^ Kruskal–Wallis; ^(b)^ Mann–Whitney; ε^2^—Epsilon squared (Kruskal–Wallis); δ—Cliff’s Delta (Mann–Whitney); “Neither, nor” denotes a neutral response; the primary caregiver is a main current provider of informal family care; the secondary caregiver is a supportive provider; prior caregiving experience covers any informal family care provided to an older adult during the previous five years.

## Data Availability

The data used to support the findings of this study will be available from the authors upon reasonable request.

## Public Involvement Statement

An address-based sampling method was used to select the sample. The survey was conducted via face-to-face interviews.

## Guidelines and Standards Statement

This manuscript was drafted according to the STROBE checklist for cross-sectional studies, which is available at https://www.strobe-statement.org/checklists/ (accessed 30 January 2026).

## Abbreviations

The following abbreviations are used in this manuscript:

AVE	Average variance extracted
CFA	Confirmatory factor analysis
CFI	Comparative fit index
CPS	Caregiver preparedness scale
CR	Composite reliability
DF	Degrees of freedom
EFA	Exploratory factor analysis
GFI	Goodness of fit index
ITC	Item–total correlation
KMO	Kaiser–Meyer–Olkin
NFI	Normed fit index
PAF	Principal axis factoring
RMSEA	Root mean square error of approximation
SD	Standard deviation
SRMR	Standardized root mean squared residual
TLI	Tucker–Lewis index
